# *In vitro* activity of ceftobiprole and comparator antibiotics against contemporary European isolates (2016–19)

**DOI:** 10.1093/jacamr/dlac030

**Published:** 2022-03-24

**Authors:** Rafael Canton, Kamal Hamed, Tatiana Wiktorowicz, Nowel Redder, Noelle Jemmely, Juan Quevedo, Anne Santerre Henriksen

**Affiliations:** Servicio de Microbiología, Hospital Universitario Ramón y Cajal, Madrid, Spain; Instituto Ramón y Cajal de Investigación Sanitaria (IRYCIS), Madrid, Spain; Basilea Pharmaceutica International Ltd, Basel, Switzerland; Basilea Pharmaceutica International Ltd, Basel, Switzerland; ADVANZ PHARMA Switzerland, Sàrl, Geneva, Switzerland; ADVANZ PHARMA Switzerland, Sàrl, Geneva, Switzerland; ADVANZ PHARMA Switzerland, Sàrl, Geneva, Switzerland; Maxel Consulting ApS, Jyllinge, Denmark

## Abstract

**Objectives:**

To evaluate the susceptibility to ceftobiprole of clinical bacterial isolates obtained from hospitalized patients in Europe.

**Methods:**

A total of 20 000 non-duplicate bacterial isolates were collected in 2016–19 from patients with documented infections at medical centres located in 17 countries in Europe. Bacterial identification was confirmed and susceptibility to ceftobiprole and comparator agents was tested using the EUCAST broth microdilution methodology and interpretive criteria by a central microbiology laboratory.

**Results:**

Of the 20 000 isolates, 10 007 (50.0%) were Gram-positive and 9993 (50.0%) were Gram-negative. The most common species was *Staphylococcus aureus* (35.0%), followed by *Streptococcus pneumoniae* (15.0%), *Klebsiella pneumoniae* (11.1%), *Pseudomonas aeruginosa* (11.0%), *Escherichia coli* (9.7%) and *Haemophilus influenzae* (3.0%). Overall, 99.7% (6981/7000) of *S. aureus*, including 99.5% (3483/3502) of MRSA, 97.8% (2941/3007) of *S. pneumoniae*, 100% (605/605) of *H. influenzae* and 76.3% (5492/7197) of Enterobacterales isolates were susceptible to ceftobiprole. Susceptibility to ceftobiprole was higher for isolates from northern and western Europe as compared with eastern and southern Europe.

**Conclusions:**

Ceftobiprole continues to exhibit potent and broad-spectrum activity against Gram-positive and Gram-negative clinical isolates from Europe, and as expected, with a slight north-to-south and west-to-east susceptibility gradient.

## Introduction

Antimicrobial resistance poses a significant threat to global healthcare systems, contributing to longer hospital stays, increased healthcare costs and increased risk of mortality.^1^ According to the European Antimicrobial Resistance Surveillance Network (EARS-Net), although the overall prevalence of MRSA in Europe has continued to decline, MRSA remains an important pathogen in several EU/European Economic Area (EEA) countries.^2^ Decreases were also noted for *Streptococcus pneumoniae* penicillin non-wild type and macrolide resistance. With regard to Gram-negative organisms, this trend is reversed with an increase of overall resistance observed in recent years.^2^ In 2019, more than half of *Escherichia coli* and more than one-third of *Klebsiella pneumoniae* isolates were resistant to at least one antimicrobial group.^2^ For several Gram-positive and Gram-negative bacterial species–antimicrobial group combinations, a north-to-south and a west-to-east gradient was evident in the EU/EEA.^2^ In general, lower percentages of resistance were reported by countries in the north of Europe and higher percentages were reported by countries in the south and east of Europe.^2^

Ceftobiprole, the active moiety of the prodrug ceftobiprole medocaril, is an advanced-generation, broad-spectrum, IV cephalosporin. It is approved in many European countries for the treatment of community-acquired and hospital-acquired pneumonia (excluding ventilator-associated pneumonia). Recently, the TARGET Phase 3 study demonstrated that ceftobiprole is non-inferior to vancomycin and aztreonam in the treatment of acute bacterial skin and soft tissue infections, in terms of early clinical response and investigator-assessed clinical success at the test-of-cure visit.^3^ Ceftobiprole is currently under Phase 3 investigation for the treatment of *Staphylococcus aureus* bacteraemia to support a new drug application in the USA.^4^

Since 2016 ceftobiprole has been included in two main surveillance programmes in Europe: the BSAC respiratory and bacteraemia surveillance^5^ and another pan-European surveillance performed by International Health Management Associates Europe (IHMA).^6^

The objective of the current study was to examine the susceptibility profiles of ceftobiprole and comparator agents tested by a standardized reference methodology against 20 000 clinical isolates collected during the years 2016 through 2019 from European region medical centres.

## Methods

### Bacterial isolates

**Figure 1.**
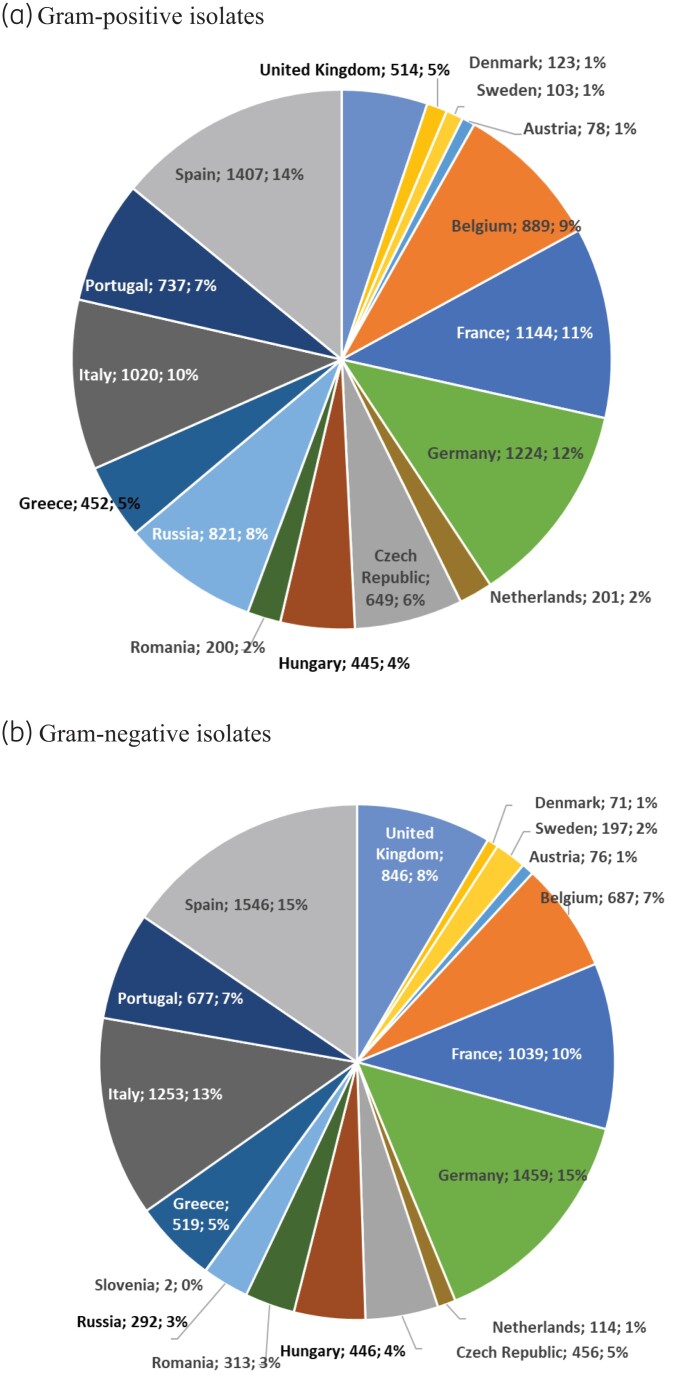
Percentage of isolates collected per country (country; *n*; %). (a) Gram-positive isolates. (b) Gram-negative isolates.

A total of 20 000 non-duplicate clinical isolates from the Ceftobiprole IHMA Surveillance Program in Europe (2016–19) were submitted from medical centres in 17 countries (number of isolates): Austria (154), Belgium (1576), Czech Republic (1105), Denmark (194), France (2183), Germany (2683), Greece (971), Hungary (891), Italy (2273), the Netherlands (315), Portugal (1414), Romania (513), Russia (1113), Slovenia (2), Spain (2953), Sweden (300) and the UK (1360) (Figure 1). All organisms were isolated from different documented infection types, and only one isolate per patient infection episode was included in the surveillance collection. Isolates were identified by IHMA Europe (Sàrl, Monthey, Switzerland) using matrix-assisted laser desorption ionization–time of flight mass spectrometry (Bruker Daltonics, Billerica, MA, USA).

### Antimicrobial susceptibility testing

MIC values were determined by broth microdilution according to International Organization for Standardization guidelines.^7^ MIC results for ceftobiprole, imipenem and levofloxacin were obtained for all clinical isolates. For *S. aureus*, MICs were also determined for cefoxitin, clindamycin, daptomycin, erythromycin, flucloxacillin, gentamicin, linezolid, penicillin, tetracycline, trimethoprim/sulfamethoxazole and vancomycin. For other organisms, additional comparator agents tested were as follows: *S. pneumoniae*: ampicillin, azithromycin, ceftriaxone, clindamycin, linezolid, penicillin, tetracycline and trimethoprim/sulfamethoxazole; *Haemophilus influenzae*: amoxicillin/clavulanic acid, ampicillin, azithromycin, ceftriaxone, cefuroxime, chloramphenicol, tetracycline and trimethoprim/sulfamethoxazole; Enterobacterales: amoxicillin/clavulanic acid, cefepime, cefoxitin, ceftazidime, piperacillin/tazobactam and trimethoprim/sulfamethoxazole; and *Pseudomonas aeruginosa*: cefepime, ceftazidime, colistin, piperacillin and piperacillin/tazobactam.

Cefoxitin MICs were used to detect phenotypic methicillin resistance in *S. aureus*. Susceptibilities to all antibiotics were interpreted using the EUCAST clinical breakpoints v11^8^ and collating susceptible (S) and susceptible at increased exposure (I) together in line with the EUCAST recommendations. When species-specific breakpoints were not available, pharmacokinetic/pharmacodynamic (PK/PD) breakpoints were applied.^8^

### Ethics

Ethics approval was not required as all *in vitro* samples were anonymized.

## Results

### Epidemiology

The *in vitro* activity of ceftobiprole and comparators was assessed in 20 000 clinical isolates: 10 007 were Gram-positive bacteria (35.0% MSSA, 35.0% MRSA and 30.0% *S. pneumoniae*) and 9993 were Gram-negative bacteria [mostly Enterobacterales (72.0%), but also included *P. aeruginosa* (22%) and *H. influenzae* (6%)] (Table 1).

**Table 1.** Frequency of occurrence and cumulative percentage distribution of ceftobiprole MICs for all European clinical isolates tested (2016–19)

Organism (number of isolates tested)	Number of isolates (cumulative %) inhibited as function of ceftobiprole MIC, mg/L									Susceptibility, %
	≤0.06	0.12	0.25	0.5	1	2	4	8	>8	
Gram-positive bacteria										
* S. aureus* (7000)	3 (0.0)	30 (0.5)	1291 (18.9)	2631 (56.5)	2043 (85.7)	983 (99.7)	19 (100)			99.7
MSSA (3498)	2 (0.1)	30 (0.9)	1278 (37.4)	2175 (99.6)	13 (100)					100
MRSA (3502)	1 (0.0)	0 (0.0)	13 (0.4)	456 (13.4)	2030 (71.4)	983 (99.5)	19 (100)			99.5
* S. pneumoniae* (3007)	2411 (80.2)	74 (82.6)	153 (87.7)	303 (97.8)	64 (99.9)	1 (99.9)	1 (100)			97.8
Penicillin susceptible (953)	953 (100)									100
Penicillin intermediate^a^ (702)	259 (36.9)	69 (46.7)	142 (67.0)	211 (97.0)	21 (100)					97.0
Penicillin resistant (148)		1 (0.7)	10 (7.4)	92 (69.6)	43 (98.6)	1 (99.3)	1 (100)			69.6
Gram-negative bacteria										
Enterobacterales (7197)	4853 (67.4)	402 (73.0)	237 (76.3)	162 (78.6)	104 (80.0)	65 (80.9)	56 (81.7)	47 (82.3)	1271 (100)	76.3
Ceftazidime susceptible^b^ (5791)	4714 (81.4)	362 (87.7)	208 (91.3)	143 (93.7)	77 (95.0)	26 (95.5)	14 (95.7)	12 (95.9)	235 (100)	91.3
Ceftazidime resistant (1406)	139 (9.9)	40 (12.7)	29 (14.8)	19 (16.1)	27 (18.1)	39 (20.8)	42 (23.8)	35 (26.3)	1036 (100)	14.8
* Enterobacter* spp. (822)	545 (66.3)	44 (71.7)	19 (74.0)	11 (75.3)	18 (77.5)	32 (81.4)	34 (85.5)	28 (88.9)	91 (100)	74.0
Ceftazidime susceptible^b^ (555)	499 (89.9)	31 (95.5)	12 (97.7)	3 (98.2)	1 (98.4)			1 (98.6)	8 (100)	97.7
Ceftazidime resistant (267)	46 (17.2)	13 (22.1)	7 (24.7)	8 (27.7)	17 (34.1)	32 (46.1)	34 (58.8)	27 (68.9)	83 (100)	24.7
* E. coli* (1948)	1558 (80.0)	45 (82.3)	25 (83.6)	8 (84.0)	2 (84.1)	1 (84.1)	3 (84.3)	4 (84.5)	302 (100)	83.6
Ceftazidime susceptible^b^ (1712)	1546 (90.3)	42 (92.8)	22 (94.0)	7 (94.5)	1 (94.5)	1 (94.6)	3 (94.7)	3 (94.9)	87 (100)	94.0
Ceftazidime resistant (236)	12 (5.1)	3 (6.4)	3 (7.6)	1 (8.1)	1 (8.5)			1 (8.9)	215 (100)	7.6
* Klebsiella* spp. (3191)	1843 (57.8)	160 (62.8)	132 (66.9)	115 (70.5)	68 (72.6)	30 (73.6)	12 (74.0)	11 (74.3)	820 (100)	66.9
Ceftazidime susceptible^b^ (2358)	1763 (74.8)	144 (80.9)	125 (86.2)	111 (90.9)	63 (93.6)	23 (94.5)	8 (94.9)	7 (95.2)	114 (100)	86.2
Ceftazidime resistant (833)	80 (9.6)	16 (11.5)	7 (12.4)	4 (12.9)	5 (13.4)	7 (14.3)	4 (14.8)	4 (15.3)	706 (100)	12.4
* K. pneumoniae* (2223)	1342 (60.4)	75 (63.7)	28 (65.0)	15 (65.7)	21 (66.6)	14 (67.3)	7 (67.6)	6 (67.8)	715 (100)	65.0
Ceftazidime susceptible^b^ (1513)	1335 (88.2)	70 (92.9)	27 (94.6)	14 (95.6)	19 (96.8)	8 (97.4)	4 (97.6)	2 (97.8)	34 (100)	94.7
Ceftazidime resistant (710)	7 (1.0)	5 (1.7)	1 (1.8)	1 (2.0)	2 (2.3)	6 (3.1)	3 (3.5)	4 (4.1)	681 (100)	1.8
* Serratia marcescens* (618)	380 (61.5)	133 (83.0)	43 (90.0)	22 (93.5)	12 (95.5)	2 (95.8)	3 (96.3)	2 (96.6)	21 (100)	90.0
Ceftazidime susceptible^b^ (602)	380 (63.1)	133 (85.2)	42 (92.2)	22 (95.8)	12 (97.8)	2 (98.2)	2 (98.5)	1 (98.7)	8 (100)	92.2
Ceftazidime resistant (16)			1 (6.3)				1 (12.5)	1 (18.8)	13 (100)	6.3
* P. mirabilis* (618)	527 (85.3)	20 (88.5)	18 (91.4)	6 (92.4)	4 (93.0)		4 (93.7)	2 (94.0)	37 (100)	91.4
Ceftazidime susceptible^b^ (564)	526 (93.3)	12 (95.4)	7 (96.6)				1 (96.8)		18 (100)	96.6
Ceftazidime resistant (54)	1 (1.9)	8 (16.7)	11 (37.0)	6 (48.1)	4 (55.6)		3 (61.1)	2 (64.8)	19 (100)	37.0
* P. aeruginosa* (2191)	2 (0.1)	2 (0.2)	5 (0.4)	48 (2.6)	257 (14.3)	700 (46.3)	422 (65.5)	311 (79.7)	444 (100)	65.5
Imipenem non-resistant (1609)	2 (0.1)	2 (0.2)	5 (0.6)	46 (3.4)	241 (18.4)	642 (58.3)	339 (79.4)	198 (91.7)	134 (100)	79.4
Imipenem resistant (582)				2 (0.3)	16 (3.1)	58 (13.1)	83 (27.3)	113 (46.7)	310 (100)	27.3
Ceftazidime susceptible^b^ (1373)	2 (0.1)	2 (0.3)	5 (0.7)	46 (4.0)	239 (21.4)	596 (64.8)	245 (82.7)	134 (92.6)	104 (100)	82.7
Ceftazidime resistant (818)				2 (0.2)	18 (2.4)	104 (15.2)	177 (36.8)	177 (58.4)	340 (100)	36.8
* H. influenzae* (605)	368 (60.8)	108 (78.7)	50 (86.9)	30 (91.9)	29 (96.7)	20 (100)				100
Ampicillin susceptible (451)	285 (63.2)	78 (80.5)	33 (87.8)	23 (92.9)	13 (95.8)	19 (100)				100
Ampicillin resistant (154)	83 (53.9)	30 (73.4)	17 (84.4)	7 (89.0)	2 (90.3)	15 (100)				100
Ampicillin susceptible, AMC resistant (41)	10 (24.4)	18 (68.3)	6 (82.9)	5 (95.1)		2 (100)				100

AMC, amoxicillin/clavulanic acid.

a Referred as susceptible, increased exposure.

b Includes susceptible plus susceptible at increased exposure.

The isolates were mostly collected at medical centres in Germany, France, Italy, Spain and the UK (57% of the isolates). Eastern Europe (Czech Republic, Hungary, Romania, Russia and Slovenia) represented 18% of the collected isolates (Figure 1). Of note, medical centres in Russia contributed many more Gram-positive than Gram-negative isolates (821 versus 292).

### Ceftobiprole results by pathogen

Ceftobiprole demonstrated high activity against *S. aureus (*6981/7000, 99.7% susceptible), including MRSA (3483/3502, 99.5% susceptible) (Table 1). Only 19 (0.5%) MRSA isolates with ceftobiprole MIC of 4 mg/L were isolated cumulatively over the last 3 years of the surveillance (2017–19). These MRSA isolates came from six different countries (Belgium, France, Germany, Hungary, Italy and Russia). Italy (7 MRSA isolates) and Russia (6 MRSA isolates) had most of the ceftobiprole-resistant MRSA isolates.

*S. pneumoniae* was largely susceptible to ceftobiprole (97.8%, 2941/3007), including 69.6% (103/148) of the penicillin-resistant *S. pneumoniae* isolates (Table 1). *S. pneumoniae* resistance to ceftobiprole ranged between 0.5% in northern Europe and 3.2% in southern Europe (Table 2).

**Table 2.** Ceftobiprole-resistant isolates from 17 countries in Europe

Pathogen	Percentage of resistant isolates (*n*/*N*) across European regions^a^			
	North	South	West	East
MRSA	0 (0/198)	0.4 (7/1611)	0.5 (5/997)	1.0 (7/696)
*S. pneumoniae*	0.5 (1/191)	3.2 (29/897)	1.5 (18/1212)	2.5 (18/707)
Enterobacterales	16.3 (127/779)	27.4 (814/2971)	19.9 (487/2442)	27.6 (277/1005)
*P. aeruginosa* ^b^	29.0 (79/272)	36.4 (299/822)	29.7 (207/696)	42.4 (170/401)
All isolates^b^	8.8 (164/1854)	12.8 (976/7611)	7.7 (532/6911)	11.1 (401/3624)

a European regions: North = Denmark, Sweden, UK; West = Austria, Belgium, France, Germany, the Netherlands; East = Czech Republic, Hungary, Romania, Russia, Slovenia; South = Greece, Italy, Portugal, Spain.

b PK/PD breakpoint was applied.

Most of the Enterobacterales were susceptible to ceftobiprole (76.3%, 5492/7197). An overall lower rate of susceptibility was reported for *K. pneumoniae* (65.0%, 1445/2223), while a higher rate was reported for *Proteus mirabilis* (91.4%, 565/618). The susceptibility rate of Enterobacterales to ceftobiprole was higher in ceftazidime-susceptible isolates (86.2%–97.7%) than in the ceftazidime-resistant isolates (1.8%–37.0%) (Table 1).

Similarly to the Enterobacterales, the activity of ceftobiprole against *P. aeruginosa* was comparable to that of ceftazidime, with a higher activity of ceftobiprole against ceftazidime-susceptible isolates (82.7%) than ceftazidime-resistant isolates (36.8%) (Table 1).

For Enterobacterales, resistance to ceftobiprole varied between 16.3% in northern Europe and 27.6% in eastern Europe. Geographical distribution of *P. aeruginosa* isolates resistant to ceftobiprole followed the same pattern (Table 2).

The highest MIC of ceftobiprole observed for *H. influenzae* was 2 mg/L and ceftobiprole MIC distribution was similar for ampicillin-susceptible and ampicillin-resistant *H. influenzae* isolates (Table 1).

In total, 2073/20 000 (10.4%) isolates had a ceftobiprole MIC >4 mg/L. These were exclusively Gram-negative pathogens mostly from southern Europe (47.1%). The percentage of isolates with MIC above the PK/PD breakpoint varied between 7.7% in western Europe to 12.8% in southern Europe (Table 2).

### Ceftobiprole versus comparators

Susceptibilities of a range of pathogens to ceftobiprole and comparator agents are presented in Table 3. Ceftobiprole, daptomycin, linezolid, trimethoprim/sulfamethoxazole and vancomycin demonstrated similar activity against *S. aureus* regardless of resistance to methicillin. Susceptibilities to clindamycin, erythromycin, gentamicin, levofloxacin and tetracycline were lower in MRSA than in MSSA. One MRSA isolate from Italy showed a vancomycin MIC of 4 mg/L; this isolate was susceptible to ceftobiprole, linezolid and trimethoprim/sulfamethoxazole, but was resistant to daptomycin (MIC >2 mg/L).

**Table 3.** Antimicrobial activities of ceftobiprole and comparator agents when tested against clinical isolates from European medical centres

Pathogen (*n*)/Antibiotic	MIC (mg/L)			MIC interpretation, % susceptible^a^
	Range	MIC_50_	MIC_90_	
*S. aureus (*7000)				
Ceftobiprole	≤0.015 to 4	0.5	2	99.7
Clindamycin	≤0.03 to >1	0.12	>1	85.3
Daptomycin	≤0.06 to >2	0.5	1	99.6
Erythromycin	≤0.12 to >4	0.5	>4	59.9
Gentamicin	≤0.06 to >2	0.25	0.5	92.3
Levofloxacin	≤0.03 to >4	0.25	>4	59.7
Linezolid	0.25 to >4	2	2	99.9
Tetracycline	≤0.06 to >4	0.25	>4	89.5
Trimethoprim/sulfamethoxazole	≤0.06 to >4	≤0.06	≤0.06	99.7
Vancomycin	≤0.25 to 4	0.5	1	99.9
MSSA (3498)				
Ceftobiprole	0.03 to 1	0.5	0.5	100
Clindamycin	≤0.03 to >1	0.12	0.12	97.3
Daptomycin	0.12 to- >2	0.5	1	99.7
Erythromycin	≤0.12 to >4	0.25	>4	80.3
Gentamicin	≤0.06 to >2	0.25	0.5	97.8
Levofloxacin	0.06 to >4	0.25	1	94.0
Linezolid	0.25 to 4	2	2	100
Tetracycline	≤0.06 to >4	0.25	0.25	95.9
Trimethoprim/sulfamethoxazole	≤0.06 to >4	≤0.06	≤0.06	99.9
Vancomycin	≤0.25 to 2	1	1	100
MRSA (3502)				
Ceftobiprole	≤0.015 to 4	1	2	99.5
Clindamycin	≤0.03 to >1	0.12	>1	73.4
Daptomycin	≤0.06 to >1	0.5	1	99.5
Erythromycin	≤0.12 to >4	>4	>4	39.3
Gentamicin	≤0.06 to >2	0.25	>2	86.9
Levofloxacin	0.06 to >4	>4	>4	25.5
Linezolid	0.25 to >4	1	2	99.9
Tetracycline	≤0.06 to >4	0.25	>4	83.1
Trimethoprim/sulfamethoxazole	≤0.06 to >4	≤0.06	≤0.06	99.4
Vancomycin	≤0.25 to 4	0.5	1	99.9
*S. pneumoniae* (3007)				
Ceftobiprole	≤0.06 to 4	≤0.06	0.5	97.8
Ampicillin	≤0.03 to >8	0.06	4	86.6
Azithromycin	≤0.03 to >8	0.06	>8	76.4
Ceftriaxone	≤0.015 to >8	0.03	1	99.5
Clindamycin	≤0.015 to >2	0.03	>2	83.9
Imipenem	≤0.015 to >2	≤0.015	0.25	99.4
Levofloxacin	≤0.12 to >8	1	1	99.5
Linezolid	≤0.12 to 2	1	1	100
Penicillin	≤0.06 to >8	≤0.06	2	95.1
Tetracycline	≤0.03 to >8	0.25	>8	79.0
Trimethoprim/sulfamethoxazole	≤0.06 to >8	0.25	8	81.1
Enterobacterales (7197)				
Ceftobiprole	≤0.06 to >8	≤0.06	>8	76.3
Amoxicillin/clavulanic acid	0.25 - >64	32	>64	43.9
Ampicillin	≤0.12 to >64	>64	>64	15.4
Cefepime	≤0.008 to >8	0.06	>8	84.8
Ceftazidime	≤0.03 to >8	0.25	>8	80.5
Gentamicin	≤0.12 to >8	0.5	>8	87.5
Imipenem	≤0.03 to >8	0.12	2	96.1
Levofloxacin	≤0.004 to >4	0.06	>4	79.3
Piperacillin/tazobactam	≤0.12 to >32	2	>32	81.3
Trimethoprim/sulfamethoxazole	≤0.12 to >4	≤0.12	>4	74.9
*P. aeruginosa* (2191)				
Ceftobiprole	≤0.06 to >8	4	>8	65.5^b^
Cefepime	≤0.06 to >32	4	32	77.9
Ceftazidime	0.12 to >32	4	32	73.3
Colistin	0.12 to >32	1	2	99.5
Gentamicin	≤0.06 to >32	2	32	8.0^b^
Imipenem	≤0.06 to >32	2	32	73.4
Levofloxacin	≤0.06 to >32	1	>32	63.9
Piperacillin	0.12 to >32	8	>32	69.1
Piperacillin/tazobactam	≤0.06 to >32	8	>32	71.9
*H. influenzae* (605)				
Ceftobiprole	≤0.06 to 2	≤0.06	0.5	100^b^
Amoxicillin/clavulanic acid	0.06 to 64	0.5	4	88.4
Ampicillin	0.06 to >8	0.5	>8	74.5
Ceftriaxone	<0.001 to >0.5	0.004	0.03	95.0
Cefuroxime	≤0.06 to >8	0.5	4	85.5
Chloramphenicol	≤0.12 to >4	0.5	0.5	98.0
Imipenem	≤0.06 to >8	0.5	2	75.1
Levofloxacin	≤0.004 to >4	0.015	0.03	95.7
Tetracycline	≤0.06 to >8	0.25	0.5	99.5
Trimethoprim/sulfamethoxazole	≤0.008 to >4	0.03	4	76.9

MIC*_n_*, MIC for *n*% of isolates tested.

a Percentage of susceptible plus susceptible at increased exposure.

b PK/PD breakpoint was applied.

Ceftobiprole, ceftriaxone, imipenem, linezolid and levofloxacin had the highest activity against *S. pneumoniae.* Only 76.2% of the *S. pneumoniae* isolates were susceptible to azithromycin.

For Enterobacterales, imipenem and gentamicin were the most active (93.6% and 87.5%, respectively). Ampicillin and amoxicillin/clavulanic acid had the lowest activity against Enterobacterales (15.4% and 43.9%, respectively). All other antibiotics tested had similar activity, ranging from 74.9% for trimethoprim/sulfamethoxazole to 84.8% for cefepime.

Colistin was the most active antibiotic (99.5%) against *P. aeruginosa*. The β-lactams tested had similar activity: 65.5% for ceftobiprole, 69.1% for piperacillin, 71.9% for piperacillin/tazobactam, 73.3% for ceftazidime, 73.4% for imipenem/cilastatin and 77.9% for cefepime. Only 63.9% of the *P. aeruginosa* isolates tested were susceptible to levofloxacin.

Ceftobiprole was highly active, similar to ceftriaxone, chloramphenicol, levofloxacin and tetracycline, against *H. influenzae*. Trimethoprim/sulfamethoxazole and ampicillin had the lowest activity against *H. influenzae* (76.9%).

## Discussion

Among healthcare-associated infections, Enterobacterales, *S. aureus*, *S. pneumoniae*, *P. aeruginosa* and *Haemophilus* species represent nearly 70% of the pathogens isolated.^9^ In particular, hospital-acquired pneumonia, which accounts for about 26% of all nosocomial infections in European acute care hospitals, can be associated with significant mortality.^9^ This large (20 000 isolates) and longitudinal (2016–19) European antimicrobial surveillance study confirmed ceftobiprole’s *in vitro* activity against these major pathogens of respiratory infections. These findings are consistent with previous reports from this surveillance program.^6,10^ An analysis of 4854 key Gram-positive and Gram-negative isolates collected in 2018 revealed potent *in vitro* activity of ceftobiprole against *S. aureus* and *S. pneumoniae* (above 98% susceptibility) and susceptibility of Enterobacterales and *P. aeruginosa* consistent with the current study (75.6% and 63.2% respectively).^10^

Only 19 (0.5%) MRSA isolates were resistant to ceftobiprole (MIC of 4 mg/L). Most of these isolates came from Italy where MRSA resistance to ceftobiprole has been reported since 2018^11,12^ and Russia where such resistance has been only recently reported.^6^ Of the three MRSA isolates resistant to ceftobiprole reported by Hawser *et al.*,^6^ two were characterized as clonal complex 8 (CC8) and the third as CC5, and different mutations were present in genes encoding penicillin-binding proteins, *mecA* and other proteins in each resistant isolate. No genomic sequencing was performed in the current study to further explore the molecular mechanisms present in MRSA isolates.

For the majority of Gram-negative bacteria, susceptibility to ceftobiprole was similar to that of third-generation cephalosporins such as ceftazidime, as reported previously.^5^

Geographical differences in the resistance to ceftobiprole appear to be consistent with overall resistance to antibiotics reported in Europe, i.e. greater susceptibility in northern and western Europe versus southern and eastern Europe.^2^

Compared with the latest reported large longitudinal study (2005–10) that investigated European isolates collected before its marketing authorization in Europe,^13^ ceftobiprole continues to exhibit potent antimicrobial activity.

The current study has several limitations. Firstly, mainly older antibiotics were tested, making it impossible to assess ceftobiprole’s activity versus that of newer comparators such as ceftaroline, eravacycline or delafloxacin. Secondly, although all isolates came from hospitalized patients with documented infections, the infection source was not recorded. This represents a gap for future studies, particularly for infections, where selecting the most effective therapy at the earliest timepoint is critical for patient survival.^14^ Finally, while the geographical spread of collected isolates was generally representative of most regions in Europe, most isolates came from southern and western Europe; northern Europe represented only 10% of the isolates collected. However, the pattern of isolate susceptibility is consistent with previously published reports by EARS-Net, with an observed north-south and west-east gradient.^2^

In summary, ceftobiprole exhibited excellent coverage of Gram-positive pathogens, including MRSA and *S. pneumoniae*, and has a spectrum of activity against Gram-negative bacilli similar to that of third-generation cephalosporins. These contemporary *in vitro* results from an extensive European surveillance study evaluating 20 000 organisms confirm a large volume of earlier reports on the broad spectrum of ceftobiprole activity.^6,10,13,15–17^ Ceftobiprole continues to be an attractive antibiotic option for the treatment of infections in which *S. aureus* (and particularly MRSA) is a concern and infections in which Gram-positive and Gram-negative pathogens may both be present.
